# What Illinois Veterinarians Need

**Published:** 1898-06

**Authors:** 


### WHAT ILLINOIS VETERINARIANS NEED.

Higher recognition of the value and worth of a diploma, and the education that such carries with it.

Better compensation for contract work and less of this done for inadequate sums, with the dependence upon the value of support of certain firms as an advertisement.

A higher recognition by the State and cities of the value of a veterinary degree, and of the better education of those with college training.

Closer community of those better-educated members of the profession, and the sustaining of the dignity of the calling on a proper basis.

Those who are best able to pay should be the ones that should lead in their compensations of veterinarians, while the single-horse owner should bear proportionately less burden.

Capital invested in livestock should not be treated from a charitable standpoint.

The State possesses two regular, recognized veterinary colleges, and these should set the pace for the advance of the veterinary profession all along the lines by the highest examples of the importance and value of a veterinary education.

More local associations over the State at centres easy of access to local members of the profession.

An increased membership in their State organization.

To increase their contributions to veterinary literature.

The Chicago Civil Service Board to be more aggressive in enforcing existing legislation as it applies to the public employment of veterinarians.

A State Board of Registration of all veterinarians. A high license fee, to afford means for vigorously enforcing such a law.

A larger membership in the U. S. V. M. A.

